# Endoscopic Retrograde Appendicitis Therapy in Subacute Appendicitis With Abscess: A Comprehensive Case Study and Innovative Insights

**DOI:** 10.7759/cureus.54087

**Published:** 2024-02-12

**Authors:** Bhuvi Raxwal, Jayashree Payappagoudar, Satish Patel

**Affiliations:** 1 Medicine, Access Health Care Physicians, Zephyrhills, USA; 2 Internal Medicine, BayCare Health System, New Port Richey, USA; 3 Gastroenterology, HCA Florida Trinity Hospital, New Port Richey, USA

**Keywords:** appendiceal stent, polypectomy, cecal intussusception, laparoscopic appendectomy, abscess, subacute appendicitis, endoscopic retrograde appendicitis therapy (erat), appendicitis

## Abstract

This case report explores the feasibility and efficacy of endoscopic retrograde appendicitis therapy (ERAT) in treating a 42-year-old female with subacute appendicitis complicated by an abscess. The patient, initially presenting with abdominal pain, underwent an endoscopic intervention involving drainage, irrigation, and stent placement in the appendix. The study discusses the patient's successful outcome, emphasizing the role of ERAT in managing complicated appendicitis with abscesses.

The report outlines the case presentation with the initial misdiagnosis of cecal intussusception. The endoscopic procedure involved identifying a partially prolapsed appendix, spontaneous drainage of purulent discharge, and subsequent stent placement to facilitate drainage while awaiting definitive surgery. The patient's positive response to ERAT was marked by pain reduction and a follow-up CT scan confirming the absence of an abscess and a normal appendix.

The case report asserts that ERAT emerges as a promising alternative to immediate appendectomy for patients with subacute appendicitis complicated by abscesses, enhancing symptom relief and reducing major adverse events.

## Introduction

Acute appendicitis is a common surgical emergency that has a lifetime risk of appendicitis of 8.6% for males and 6.7% for females [1,2]. Laparoscopic appendectomy remains the standard treatment. Uncomplicated acute appendicitis can be safely treated with antibiotics, with a recurrence rate of approximately 39% at five-year follow-up [3].

Endoscopic retrograde appendicitis therapy (ERAT) is an emerging endoscopic procedure for the management of patients with acute appendicitis with high rates of clinical and technical success with a recurrence rate of 6% [4].

Drawing inspiration from endoscopic retrograde cholangiopancreatography (ERCP) technology, this method utilizes an endoscope equipped with a transparent cap at its distal end. The procedure involves intubating the appendix to decompress the lumen, followed by complete drainage of the appendix cavity using the Seldinger technique. Direct endoscopic imaging or fluoroscopic endoscopic retrograde appendicography (ERA) is employed to differentiate between suspected and confirmed acute appendicitis.

This case report presents a case of subacute appendicitis complicated by an abscess in a 42-year-old female patient and discusses current management strategies.

## Case presentation

A 42-year-old Asian woman presented with mild abdominal cramping pain (3/10 in intensity) in the lower quadrant, non-radiating with no rebound or rigidity on the exam. The pain has been intermittent, fluctuating over a week, and not associated with eating. She reported no fever, chills, constipation, or diarrhea. The patient denied any weight loss in the past year. The patient has not had any similar episodes in the past and no previous abdominal surgeries. There was no family history of gastroenterological diseases or cancer. The patient denied any history of smoking, alcohol, or drug use.

The patient had a past medical history of Grave's disease treated with methimazole. She was empirically prescribed broad-spectrum antibiotics (amoxicillin and metronidazole).

**Figure 1:**
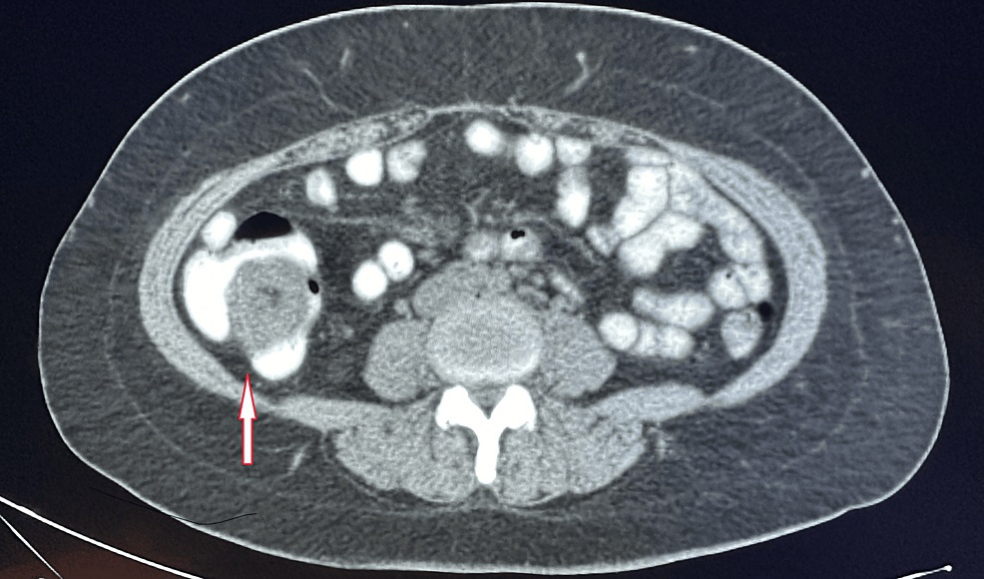
CT scan of the abdomen An arrow pointing to the cecal intussusception.

A CT scan of the abdomen and pelvis revealed cecal intussusception with non-visualization of the appendix (Figure 1). She remained afebrile with a normal white blood cell count.

**Figure 2:**
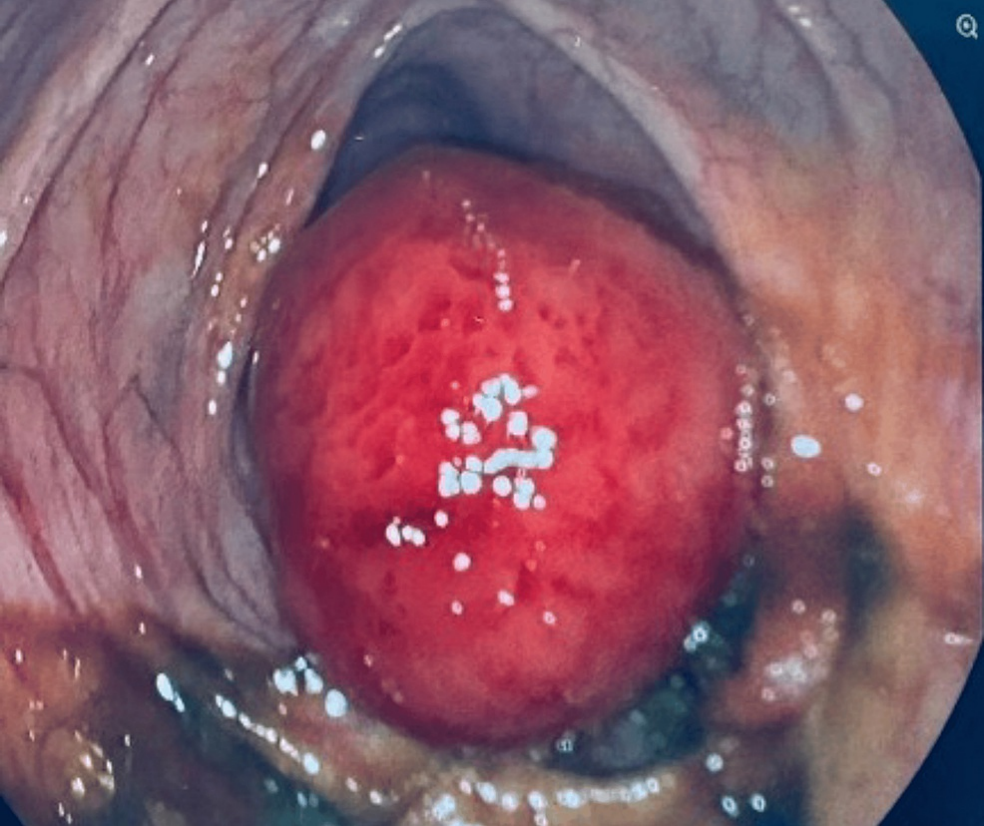
Diagnostic colonoscopy demonstrating polypoid mass

The patient underwent a colonoscopy, which exhibited a large polyp (Figure 2). The biopsy of the polyp revealed normal tissue without any atypical cells suggestive of malignancy. The patient was recommended for a polypectomy and was referred to a tertiary care center.

Colonoscopy and intervention

At the Mayo Clinic, a subsequent colonoscopy and intervention uncovered purulent discharge at the appendix's base. The treatment plan underwent thorough discussion with the surgical team, resulting in the decision to proceed with ERAT, followed by antibiotics and follow-up imaging to assess the response to the therapy.

**Figure 3:**
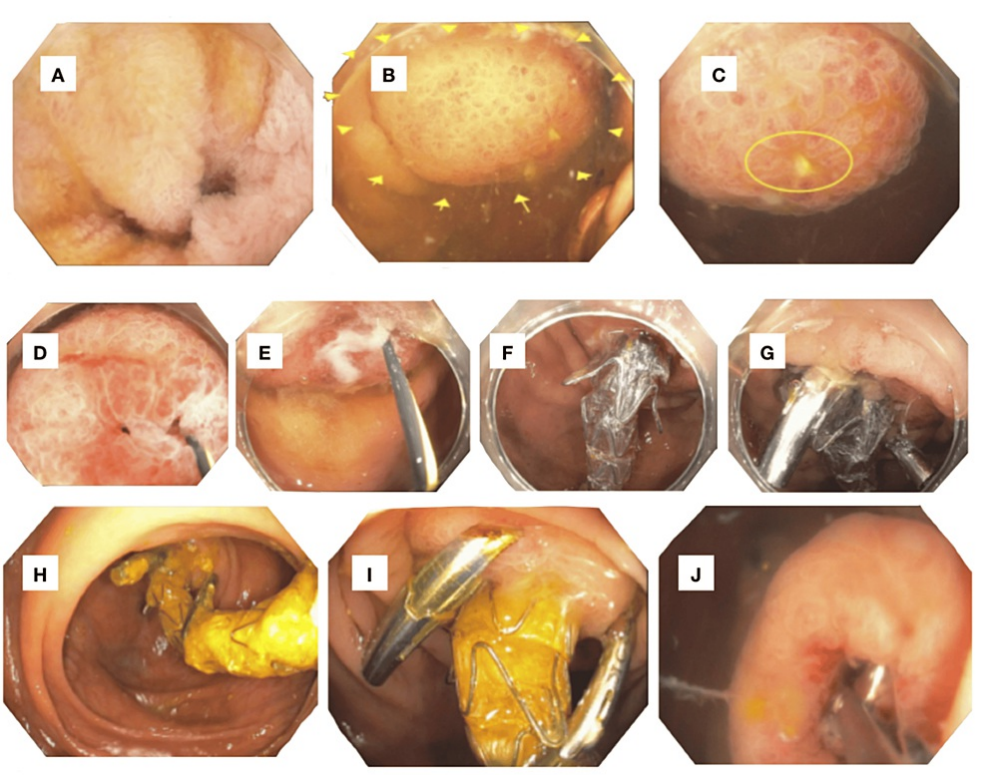
Endoscopic retrograde appendicitis therapy A: Ileocecal valve. B: Cecum - partially prolapsed appendix. C: Appendiceal orifice with pus. D: Appendiceal orifice - guidewire into the appendiceal orifice. E: Appendiceal orifice - guidewire into the appendiceal orifice with spontaneous drainage of pus. F: Appendiceal orifice - placement of fully covered Viabil stent into the appendiceal orifice. G: Appendiceal orifice - placement of three instinct clips to anchor the Viabil stent into the appendiceal orifice. H: Appendiceal orifice - previously placed Viabil stent protruding out of the appendiceal orifice. I: Appendiceal orifice - previously placed Viabil stent protruding out of the appendiceal orifice. J: Appendiceal orifice after removal of the Viabil stent.

A large polypoid lesion with mucosal hyperemia was seen at the site of the appendiceal orifice. Upon further investigation, this was a partially prolapsed appendix into the lumen of the cecum, with spontaneous drainage of pus from the swollen appendiceal orifice. Interrogation of the appendiceal orifice with a Jagwire resulted in spontaneous drainage of 5 ml of purulent discharge, and endoscopic appendiceal irrigation was performed; no appendiceal fecalith was noted. After placement of a Jagwire to confirm the appendiceal orifice, an 8 mm x 6 cm fully covered metal stent (Viabil, Gore Medical, Flagstaff, AZ) was placed under fluoroscopic guidance into the appendiceal orifice for drainage of the appendix and anchored into place with three instinct clips to prevent stent outmigration while awaiting definitive surgical intervention (Figure 3).

**Figure 4:**
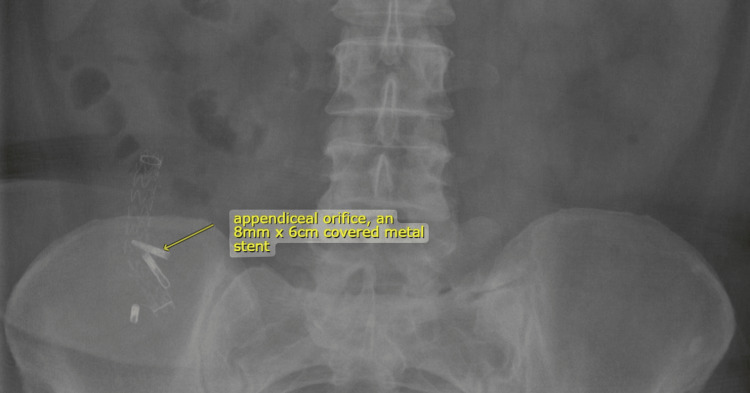
X-ray of the abdomen demonstrating appendiceal stent

The patient was initiated on antibiotics for two weeks with ciprofloxacin and metronidazole. The patient reported visual analog scale values of <3 for pain at six hours after treatment. The patient remained afebrile; repeat blood work showed a normal white blood cell count. The patient had a repeat CT of the abdomen and pelvis that showed a normal appendix with no notable abscess. The patient had a follow-up abdominal X-ray that showed the stent in place (Figure 4).

After four weeks, the stent was removed with no notable discharge. Further definitive treatment, namely, an appendectomy, was planned based on the findings from the ERAT and follow-up evaluations.

The patient successfully underwent a laparoscopic appendectomy in an outpatient setting and was discharged the same day. No significant adverse events were noted with ERAT with the stent. Post-stenting abdominal pain was noticeably improved following the procedure. The main adverse events associated with ERAT were recurrent abdominal pain.

Surgical pathology - appendix

**Figure 5:**
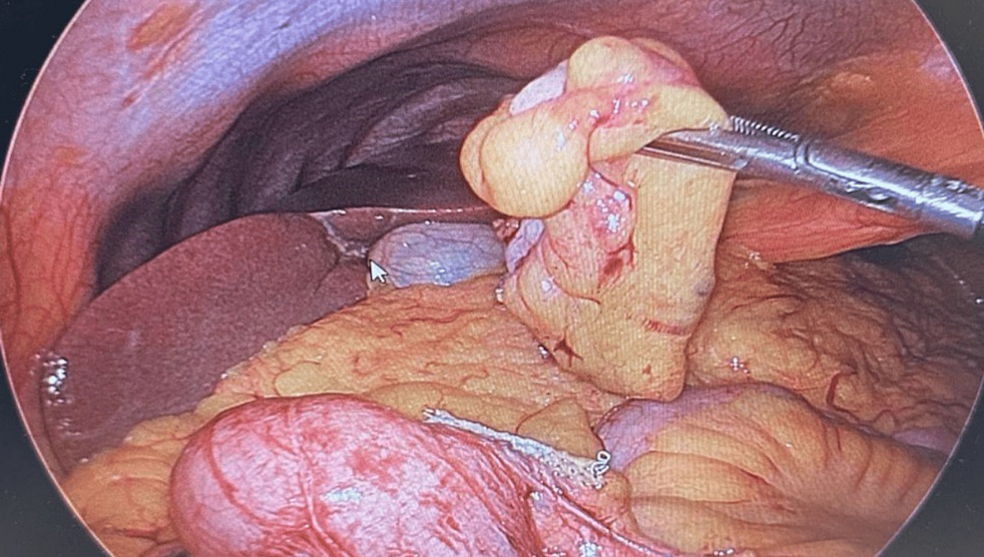
Perioperative - normal appendix

The serosa of the appendix exhibited a smooth surface without any perforation (Figure 5).

No fecalith was present within the lumen. The frozen surgical pathology results were negative, indicating the absence of malignancy.

ERAT with stent placement may offer a less invasive alternative to immediate surgery, potentially reducing the economic burden associated with a traditional appendectomy. It allowed for a staged approach, with definitive surgical intervention after initial drainage and antibiotic therapy. The successful outcome without significant adverse events suggests that ERAT with a stent was a reasonable and cost-effective approach in this case, avoiding potential complications associated with immediate surgery.

## Discussion

Acute appendicitis continues to be a prevalent surgical emergency, with laparoscopic appendectomy being the established standard of care. However, recent studies have demonstrated that in the treatment of appendicitis, antibiotics were non-inferior to appendectomy and may be considered with the patient with shared decision-making [5,6].

Managing patients with appendicitis associated with complications such as phlegmon or abscess poses considerable challenges. In this context, ERAT emerges as a valuable intervention for addressing complicated appendicitis with phlegmon or abscess with stent placement after endoscopic appendiceal irrigation (EAI) when lumen stenosis of the appendiceal, excessive pus, or appendiceal fecalith is identified [7].

In the past, patients encountering complicated appendicitis with an abscess were historically given two primary recommendations: either initial treatment with antibiotics, with a bridge to surgery, or immediate surgical intervention involving abscess drainage and antibiotics. However, contemporary evidence indicates that surgical treatment for patients presenting with appendiceal phlegmon or abscesses is more effective than antibiotic therapy alone [3].

For the management of appendiceal abscesses, attempting ERAT has shown positive outcomes, eliminating the immediate necessity for surgery. The inclusion of stent placement in ERAT can effectively eliminate appendiceal obstructions that contribute to the inflammatory process, thereby diminishing the risk of further complications. ERAT proves particularly advantageous for patients with appendicoliths, as it facilitates the flushing out of these obstructions from the appendiceal lumen. This not only alleviates symptoms but also contributes to a decreased rate of recurrent appendicitis [7]. The main adverse events associated with ERAT were spontaneous discharge of the stent, recurrent abdominal pain, and recurrent appendicitis.

ERAT poses potential challenges compared to laparoscopic appendectomy. This is particularly evident in the requirement for bowel preparation, involving the use of 2 liters of polyethylene glycol electrolyte solution. This process can be demanding for patients already experiencing abdominal pain due to appendiceal inflammation, potentially accompanied by symptoms like fever and nausea.

Combining ERAT with a brief course of antibiotics and postponing appendectomy offers a hybrid approach that has the potential to minimize hospitalization duration and diminish the necessity for readmissions. Integration of ERAT into clinical practice represents a practical strategy that could significantly reduce both immediate and prolonged adverse events [8].

ERAT is not universally applicable to all appendicitis cases, typically being reserved for uncomplicated situations without extensive inflammation. As observed in the presented case, a primary adverse event linked to ERAT is the occurrence of recurrent abdominal pain. Potential complications related to stent placement, including migration, perforation, or obstruction, are infrequent but exist. The resource-intensive nature of ERAT, demanding specialized equipment and expertise, may limit its accessibility in various healthcare settings. Delayed surgical intervention, a consequence of the staged approach, may lead to prolonged symptomatology and an increased risk of complications. Additionally, the long-term outcomes of ERAT compared to traditional appendectomy remain insufficiently established.

In managing complicated appendicitis with abscesses, a multidisciplinary approach involving interventional endoscopy, interventional radiology, and minimally invasive surgery should be considered. The choice of intervention depends on the disease's stage, aiming to minimize long-term complications.

## Conclusions

In summary, this case report highlights the viability and effectiveness of ERAT as a compelling alternative for managing subacute appendicitis with complications in a 42-year-old female. The patient's initial presentation of mild abdominal pain, lacking typical appendicitis symptoms, initially led to the misdiagnosis of cecal intussusception. ERAT, involving endoscopic drainage, irrigation, and stent placement in the appendix, demonstrated success by addressing a partially prolapsed appendix with spontaneous drainage of purulent discharge. The strategic placement of a fully covered metal stent facilitated continuous drainage, resulting in pain reduction and imaging confirmation of an absence of abscess and a normalized appendix.

The case report underscores ERAT as a promising alternative to immediate appendectomy, offering enhanced symptom relief and reducing major adverse events. The integrated multidisciplinary approach involving endoscopy, radiology, and minimally invasive surgery allows for tailored responses, minimizing long-term complications. Despite potential challenges, such as the need for bowel preparation, an integrated approach combining ERAT with antibiotics before appendectomy stands as a comprehensive and patient-centric management option, potentially reducing hospitalization duration. ERAT's success, in this case, signifies a transformative intervention for complicated appendicitis with abscesses, warranting further studies and broader clinical application to solidify its role in evolving appendicitis management.
